# New horizons for natural killer cell research in cancer, infection and inflammation

**DOI:** 10.1002/cti2.1275

**Published:** 2021-04-28

**Authors:** Fernando Souza‐Fonseca‐Guimaraes

**Affiliations:** ^1^ University of Queensland Diamantina Institute The University of Queensland Woolloongabba Qld Australia

## Abstract

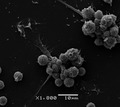

Since the discovery and description of natural killer (NK) cells, approximately 45 years ago by Rolf Kiessling^1^, ^2^ and Ronald B Herberman,^3^, ^4^ and the subsequent immunotherapy renascence era, the interest in NK cell‐based immunotherapy has dramatically increased over the last 5 years. Immune ‘checkpoint’ inhibitors represent the most significant advance in cancer treatment in the last 30 years. However, their effectiveness is limited to tumors with infiltrating tumor‐specific cytotoxic T lymphocytes, where resistance to checkpoint inhibition has been commonly observed.^5^ Natural killer (NK) cells are the innate equivalent to cytotoxic T lymphocytes and are able to spontaneously detect and kill transformed cells, thus contributing to cancer immune surveillance. Mature, functional NK cells are frequent in blood and lymphoid organs, yet cancerous cells are still able to efficiently evade NK cell detection, resulting in metastases in vital organs that ultimately lead to death.^6^ Key advances are thus required in order to target NK cells effectively to tumors. Despite these unknowns, NK cell‐based therapies are highly desirable as they are predicted to elicit fewer side effects than their T‐cell counterparts (e.g. graft‐versus‐host disease [GvHD] following allogeneic transplantation and cytokine response syndrome [CRS]). NK cell‐based therapies also do not require prior sensitisation or clonal expansion.^6^ Consequently, NK cells have been the focus of intense research leading to remarkable translational progress over the past 5 years.

This Special Feature of *Clinical & Translational Immunology (CTI)* is a collaboration amongst leading scientists from the fields of NK cell biology, tumor immunity, clinical translation, inflammation and vaccinology to expound the recent discoveries of novel regulatory mechanisms for NK cell activity and how targeting these pathways could revolutionise the treatment of a variety of diseases. Collectively, this series of articles provides an updated overview of the emerging roles for NK cells and this fascinating cellular target for immunotherapy (Figure 1).

**Figure 1:**
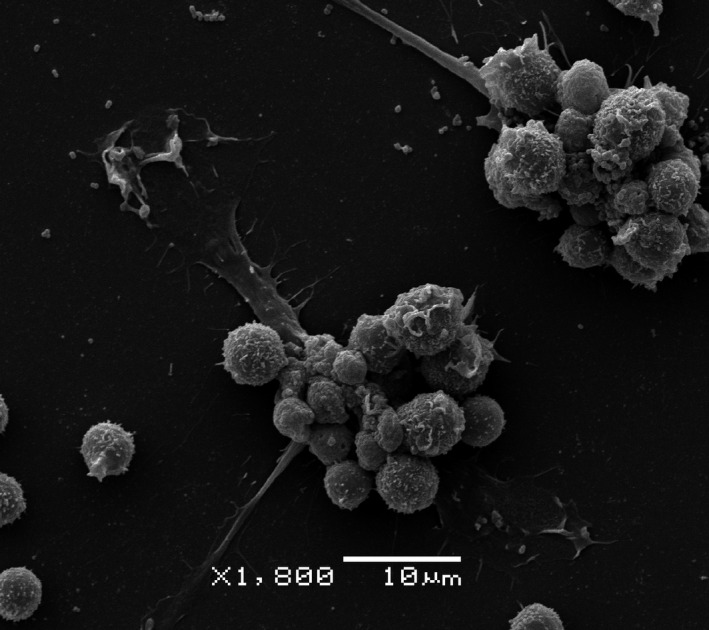
A scanning electron microscopy (SEM) image of NK cells (round cells) adhering to melanoma cells (attached/spread cells), generated at the Centro de Microscopia Electronica (CME) of the Federal University of Parana (UFPR), with the kind support of Professors Lucelia Donatti and Carolina de Oliveira, Curitiba, Brazil.

Opening this CTI Special Feature, Xing and Ferrari de Andrade provide an synopsis of a recent and unexpected mechanism by which cancer or infected cells are able to hijack NK cell‐mediated immunosurveillance.^7^ NK cells take advantage of multiple activating receptors to sense unusual cellular alterations, where this recognition is canonically regulated by the NK group 2D (NKG2D)‐activating receptor. NKG2D detects the expression of stress ligands such as the MHC class I polypeptide‐related sequence A (MICA) or MICB, which are upregulated on the cell surface in response to the cellular stress associated with tumorigenesis or viral infections. Previous work by the group^8^, ^9^ demonstrated that MICA/MICB shedding by cancer cells provides a strategy for immunoevasion from NK cell surveillance and that therapeutics preventing this shedding mechanism are a novel immunotherapy approach to restore NKG2D‐mediated surveillance. Thus, determining further strategies to prevent the shedding of these ligands is an exciting new research niche in immunotherapy with untapped potential for maximising NK cell responses against altered cells.

In addition to the evasion of NK cell‐related activation receptors, the tumor microenvironment (TME) also takes advantage of multiple inhibitory mechanisms that influence NK cell recruitment effector functions and tumoricidal function. The contribution by Riggan *et al*.^10^ highlights that not only the balance of NK cell maturation stages but also the specific release of chemokines that boost the trafficking of effective cytotoxic subsets is also key factor for efficient anticancer immunity. However, multiple suppressive pathways can be used by the TME to further prevent NK cell function, such as the TGF‐β superfamily, hypoxic conditions, prostaglandin E_2_, extracellular metabolites and intracellular checkpoints of NK cell function. Their article reviews strategies to overcome these limiting factors, which range from potential NK cell enhancement by cytokine or antibody therapies, and also outlines how novel CRISPR‐Cas9 gene editing technologies^11^, ^12^ could personalise and maximise primary NK cell responses for adoptive cancer therapies.

Translating engineered NK cell therapies to humans, Rezvani and colleagues recently performed clinical trial infusions for the first time of NK cells from cord blood transduced for expressing a CD19‐CAR construct into B‐cell leukaemia patients.^13^ Results of this trial suggest that CAR‐NK cell therapies can be safer than CAR T cells, triggering a scientific renascence of NK cells as a new frontier for cellular immunotherapies and potential off‐the‐shelf products. More recently, the group has shown that NK cells can be further engineered by CRISPR‐Cas9 to abrogate NK cell checkpoint inhibitors such as CIS, increasing cellular metabolism capacity and further enhancing their tumoricidal capacity by overcoming immunosuppression.^14^ In this Special Feature of CTI, Rezvani and colleagues highlight how the CAR‐NK cells are the *next wave of cellular therapies for cancer*.^15^ Differences, advantages and limitations versus CAR T cells are discussed together with examples from preclinical and clinical studies that use engineered NK cells for enhanced antitumor responses.

A new appreciation for vaccine development strategies in order to treat infectious diseases has emerged during the recent COVID‐19 pandemic events.^16^ Excitingly, the development of these strategies may also prove to have important applications for treating cancer. In this incredibly fast‐moving field, the success of upcoming vaccination approaches depends on continuous methodological improvement, as well as knowledge of the immune system and its response against a variety of pathologies. Previous evidence showed that NK cells are activated against several different infectious pathogens, and depend on myeloid‐derived IL‐12, IL‐18 and IFN type I signalling, which in turn contributes to T‐cell adaptive responses.^17^ Here, the NK cell and vaccination experts, Goodier and Riley, revisit the biological regulation of human NK cells by pathogens and vaccines, and discuss how these events influence NK cell differentiation, thus causing adaptation of their effector functions.^18^

Moving from vaccination to inflammation, an important aspect of NK cell biology is the additional pro‐inflammatory effector function of NK cells, which can be the first and major producers of pro‐inflammatory cytokines such as IFN‐γ and GM‐CSF.^19^ Although the appropriate secretion of such cytokines can be important to restore immunity and resolve infection, it can also contribute to exacerbated disease or overzealous/deleterious inflammation. Recently, it was discovered that NK cell‐derived GM‐CSF is a major contributor to inflammatory rheumatoid arthritis, demonstrating for the first time the participation of this innate lymphocyte in autoimmune events.^20^ These aspects, and the potential mechanisms and participation of NK cells in other inflammatory autoimmune diseases, are discussed in depth in the review by Yang and colleagues to conclude this CTI Special Feature.^21^ The role of NK cells in autoimmune inflammatory disorders is an important factor, as over‐activation or enhancing of NK cell effector functions should be taken into consideration in the context of NK cell‐targeted therapeutics, such as the treatment of cancer patients who concomitantly suffer from rheumatoid disease.

## CONFLICT OF INTEREST

FSFG is a consultant and has a funded research agreement with Biotheus Inc.
